# Reply to: Concerns regarding interpretation and reporting in Kolivas et al. mHealth low carbohydrate dietary intervention in T2D

**DOI:** 10.1038/s44324-026-00104-6

**Published:** 2026-04-24

**Authors:** Despina Kolivas, George Moschonis

**Affiliations:** 1 School of Allied Health, Human Services & Sport, La Trobe University, Bundoora, VIC Australia; 2 La Trobe Institute for Sustainable Agriculture & Food (LISAF), La Trobe University, Melbourne, VIC Australia

**Keywords:** Diseases, Endocrinology, Health care, Medical research

We provide a summary of clarifications to address issues identified in our article “mHealth low carbohydrate dietary intervention ameliorates glycaemic profile, blood pressure and weight status in people with type 2 diabetes^1^. We seek to rectify the comparison with the T2Diet study, to maintain scientific accuracy. In addition, clarifications of the secondary categorical analysis conducted with regards to (i) adherence and (ii) weight loss are presented.

*Critique: In contextualising the findings, the authors cite the T2Diet Study*^2^*, the only randomised controlled trial of a digital low carbohydrate dietary program for T2D conducted in Australia. They accurately reported the between-group differences of the 16-week intervention—HbA1c* − *0.6%, body weight* − *3.3* *kg, BMI* − *1.1* *kg/m², and reduced medication requirements—but then misrepresent these results as within-group changes, stating that “while the average reduction in HbA1c in the T2Diet study (−0.6%) was less than the improvement recorded in the current trial (−1.0%)…” (p. 6). This comparison understates the T2Diet findings and exaggerates the effect of the current intervention. In fact, the T2Diet within-group changes were HbA1c* − *0.94%, weight* − *4.36* *kg, and BMI* − *1.48* *kg/m²—all statistically significant. Distinguishing between-group differences from within-group changes is essential for fair comparison and accurate scientific and clinical interpretation*^3,4^*. Being a single-arm trial reporting within-group changes, the manuscript should clearly reflect this distinction to prevent misrepresentation of relative intervention effects*.

Response: Regarding the comparison of the primary outcome HbA1c with the T2Diet study, we acknowledge that our original statement may have inadvertently emphasised between-group differences rather than within-group changes. This statement was unintentional, and the information erroneously misinterpreted the between-group and within-group differences. We would like to thank you for highlighting this and agree that a clearer and more accurate representation would enhance the integrity of our manuscript. Upon revisiting the T2Diet publication, we confirm that the within-group reductions reported for the intervention group were indeed 0.94% for HbA1c, 4.36 kg for body weight, and 1.48 kg/m² for BMI, all statistically significant. We therefore propose the following minor correction to the relevant statement in our discussion section:

*Original: “Irrespective of these differences, the T2Diet study found over a 4 month period those provided with the web based LCD intervention in combination with standard care, significantly reduced HbA1c by 0.6%, body weight by 3.3* *kg, BMI 1.1* *kg/m*^*2*^ *and the use of diabetes medications, compared to those receiving standard care alone* 34. *While the average reduction in HbA1c in the T2Diet study trial (−0.6%) was less than the improvement recorded in the current trial (−1.0%), this provides further support for the use of LCD apps within the context of the Australian health care system.”*

Revised*:* “Irrespective of these differences, the T2Diet study found over a 4 month period those provided with the web based LCD intervention in combination with standard care, significantly reduced HbA1c by 0.94%, body weight by 4.36 kg, BMI 1.48 kg/m^2^ and the use of diabetes medications 34. The average reduction in HbA1c in the T2Diet study trial ( − 0.94%) was similar to the improvement recorded in the current trial ( − 1.0%), providing further support for the use of LCD apps within the context of the Australian health care system.*”*

This revision aims to ensure a more appropriate contextualisation of our findings and a more accurate comparison between studies.

*Critique: In several parts of the manuscript, the authors present statistically non-significant findings as significant, creating potential for misinterpretation. For example, HbA1c changes between predefined carbohydrate intake groups (≤50* *g versus* > *50* *g/day) were assessed. The results on adherence (p. 4) reported a non-significant HbA1c reduction of* − *0.5% (95% CI:* − *1.0 to 0; p* = *0.073;* Supplementary Table S3*), suggesting no association between the level of carbohydrate intake and HbA1c reduction in the cohort. However, the report then contradicts this finding by stating that “participants who reduced their HbA1c…significantly reduced their dietary carbohydrates when grouped by intake of 50* *g a day or less…(p* = *0.036)”—with no additional supporting data. Likewise, multiple statements in the abstract (p. 1), discussion (p. 5), limitations and conclusion (p. 8), described this outcome as a significant finding, implying that participants who consumed a lower carbohydrate intake had greater reductions in HbA1c—claims that do not appear to be supported by the reported analysis. Such discrepancies contravene recommended reporting practices, which emphasise that non-significant results should be described transparently and not implied to show benefit*^3–5^.

Response: To contextualise the background to our analysis, the Defeat Diabetes Program initially recommends to limit carbohydrate intake to less than 50 g per day, with the implication that over time, a unique carbohydrate tolerance, based on individual circumstances and degree of glucose impairment can be determined, as stated in the study protocol^2^. Consequently, analysis of the clinical outcomes using this limit to dietary carbohydrate, sought to understand the application of this recommendation within the study cohort to provide an objective measure of adherence after 3 months of intervention. Post-hoc chi-square analyses were conducted to examine changes from baseline in categorical outcomes, including glycaemic control status, body weight change category, achievement of clinically meaningful weight loss ( ≥ 5%), and adherence to the Defeat Diabetes dietary recommendation of limiting initial carbohydrate intake to less than or equal to 50 g/day. All variables were operationalised as binary categorical measures. Additional chi-square analyses were performed to assess the association between adherence to a carbohydrate intake of less than or equal to 50 g/day and categorical improvement in the primary outcome (HbA1c), in accordance with the Defeat Diabetes Program recommendations at intervention commencement. However, it is also important to recognise that this recommendation does not imply compliance to a strict carbohydrate limit and time duration, as it is well established that many people will exhibit an improvement in glycaemic control by simply reducing the amount of carbohydrates overall, with the degree of improvement depending on individual circumstances^3^. Thus, reductions in dietary carbohydrate from baseline overall, are more likely the driver of improved glycaemic control which may or may not be under the 50 g a day limit. As such our results showed that there was a mean decrease in HbA1c (−0.5%), and significant reduction in body weight (−2.1 kg, *p* = 0.003) in those participants who reported adherence to the 50 g a day or less carbohydrate limit, as determined by food records after 3 months of intervention. Although the improvement in glycaemic control was not found to be statistically significant, this related only to the categorical analysis with respect to adherence, which sought to understand the impact of the 50 g a day limit (i.e., the degree of carbohydrate reduction), outlined in the study protocol, and which is clearly stated in the Results and in the Discussion. The lack of statistical significance may be due to the lesser number of participants that fit within that category, which reduces the statistical power. We also highlighted in the Discussion, the inherent limitations of using food records to assess dietary patterns, that can change over time and are expected to change as part of the Defeat Diabetes Program recommendations. Not withstanding, a 0.5% reduction in HbA1c is still considered to be clinically meaningful (even without reaching statistical significance), this finding is supported by the RACGP recommendation that treatment targets for HbA1c be individualised to patient circumstances^4^. In addition, it is well established that improvement in glycaemic control, as measured by HbA1c is associated with lowering cardiometabolic risk^4–7^. We have fully disclosed this analysis, and results, and have shown the subgroup analysis for the full cohort for the reduction of dietary carbohydrate to less than 50 g a day, in Table S3.

However, to avoid misrepresentation of our results we propose the following amendment to the Discussion section.

*Original: “In addition, in terms of adherence, participants who consumed 50* *g of carbohydrate a day or less also had a greater reduction in HbA1c (−0.5%) compared to those who did not.”*

Revised: “In addition, in terms of adherence, participants who consumed 50 g of carbohydrate a day or less also had a greater nonsignificant reduction in HbA1c (−0.5%) compared to those who did not.”

To further examine adherence to the Defeat Diabetes recommendations, participants were categorised according to whether they demonstrated an improvement in glycaemic control (defined as a reduction in HbA1c from baseline) or not. Among participants who reported reducing carbohydrate intake to less than or equal to 50 g/day, 95% showed an improvement in HbA1c after 3 months of intervention. In contrast, among those who did not report reducing carbohydrate intake below 50 g/day, 80% demonstrated an improvement in HbA1c over the same period (*p* = 0.036).

Notably, the majority of participants in the cohort achieved improvements in glycaemic control through overall reductions in carbohydrate intake, with the magnitude of improvement varying according to individual circumstances. However, a significantly greater proportion of participants adhering to a carbohydrate intake of less than or equal to 50 g/day experienced improvements in glycaemic control compared with those who did not meet this threshold.

*Critique: Similarly, weight outcomes are misinterpreted. In the trial’s secondary outcome analysis for anthropometric markers—BMI, waist circumference, and body weight did not change significantly in the full sample (n* = *99; p* = *0.200). Nonetheless, the authors conducted a post-hoc subgroup analysis with 92 participants stratified by* ≥ *5% versus* < *5% weight loss (*Supplementary Table S2*). Conducting a subgroup analysis when the overall result is non-significant, especially with unexplained exclusion of 7 participants, is unusual unless such analyses were pre-specified, which does not appear to be outlined in the study methods or trial protocol*^6^*. While the authors reported the majority of participants numerically lost weight (89%), the overall intervention effect was non-significant. It is recognised that a modest weight loss of 5–10% can provide clinical benefits in the management of T2D*^7^*. However, only around one third of participants achieved* ≥ *5% weight loss. Therefore, emphasising these subgroup outcomes in the conclusion as a “clinically significant reduction in body weight” (p. 8) is misleading and risks overstating intervention efficacy to clinicians and patients. Recommended reporting practices caution that the use of post-hoc subgroup analyses should be clearly distinguished from primary outcomes and interpreted with caution to avoid giving a false impression of treatment effect*^3–5^.

Response: The study protocol provides an outline to the statistical analysis methodology employed on the outcomes examined, including the analysis of secondary outcomes and the use of categorical analysis to examine the impacts of the intervention^2^. Since our study did not include a control group, we conducted subgroup analyses to gain additional insight into how the intervention may have affected secondary outcomes in participants with different characteristics.

To address the critique regarding weight loss and the intervention effect, while subgroup analyses indicated meaningful weight loss among a subset of participants, the overall mean change in body weight across the cohort did not reach statistical significance and was openly reported. We respectfully disagree that conducting a post-hoc analysis lacks value, especially since the intervention inherently required engagement in behaviour change, and it is well established that greater adherence to carbohydrate reduction yields more pronounced improvements in glycaemic control^3,8^. Our analysis also sought to understand sex specific differences, specifically related to clinical outcomes. In performing a categorical analysis, we found that there was clinically meaningful weight loss (as defined by more than 5% of body weight) measured in about a third of study participants overall and was associated with a greater mean change in HbA1c. Furthermore, our analysis showed consistent reductions in total energy and carbohydrate intake, which aligned with observed improvements in glycaemic parameters despite minimal overall weight change. This finding aligns with evidence showing that enhanced glycaemic control can occur independently of substantial weight loss in low-carbohydrate interventions^9^.

The data collected in this study consisted of routine measurements recorded by general practitioners (GPs) as part of standard type 2 diabetes monitoring. Accordingly, the study protocol was designed to facilitate broad participation of both GPs and study participants. As some participant data were not collected by GPs or forwarded to the research team, statistical analyses were performed only on the data available. Consequently, the study followed a pragmatic design that reflects real-world clinical practice and its inherent limitations. There was no intention to mislead readers or introduce bias in the results. The limitations related to data collection and recording have been comprehensively addressed in the Discussion section of the manuscript.

Thank you again for your helpful feedback which has helped to clarify the results and interpretation of our paper and to inform our future research direction.

## Supplementary information

Supplementary information

## Data Availability

The data sets used and analysed during this study will be available from the corresponding author G.M. on reasonable request.
